# Clinical Effects and Safety of Electroacupuncture for the Treatment of Poststroke Dysphagia: A Comprehensive Systematic Review and Meta-Analysis

**DOI:** 10.1155/2020/1560978

**Published:** 2020-09-26

**Authors:** Jinke Huang, Yao Shi, Xiaohui Qin, Min Shen, Manli Wu, Yong Huang

**Affiliations:** ^1^The Second Clinical Medical College, Guangzhou University of Chinese Medicine, Guangzhou, Guangdong 510120, China; ^2^Department of Neurology, Guangdong Provincial Hospital of Chinese Medicine, Guangzhou, Guangdong 510120, China; ^3^School of Traditional Chinese Medicine, Southern Medical University, Guangzhou, Guangdong 510515, China

## Abstract

**Objectives:**

Electroacupuncture (EA), an extension of acupuncture, which is based on traditional acupuncture combined with modern electrotherapy, is commonly used for poststroke dysphagia (PSD) in clinical treatment and research. However, there is still a lack of sufficient evidence to recommend the routine use of EA for PSD. The aim of this study was to assess the efficacy and safety of EA in the treatment of PSD.

**Methods:**

Randomized controlled trials (RCTs) evaluating the effects of EA on PSD were identified through a comprehensive literature search of the PubMed, Embase, Cochrane Library, Web of Science, Chinese National Knowledge Infrastructure, Chinese Biomedical Database, and VIP databases from their inception to July 2020. The quality assessment of the included trials was performed based on the guidance of the Cochrane Reviewers' Handbook, and meta-analysis (MA) was performed by using the RevMan 5.3 software.

**Results:**

Sixteen trials were identified, and these included 1,216 patients with PSD. The results demonstrated that EA in combination with swallowing rehabilitation training (SRT) was significantly superior to SRT alone with regard to effective rate (OR 5.40, 95% CI [3.78, 7.72], *P* < 0.00001, water swallow test (WST) (MD −0.78, 95% CI [−1.07, −0.50], *P* < 0.00001), the video fluoroscopic swallowing study (VFSS) (MD 1.47, 95% CI [1.11, 1.84], *P* < 0.00001), the Ichiro Fujishima Rating Scale (IFRS) (MD 1.94, 95% CI [1.67, 2.22], *P* < 0.00001), and the incidence of aspiration pneumonia (IAP) (OR 0.20, 95% CI [0.06, 0.61], *P*=0.005).

**Conclusions:**

The results showed that EA was better than the control treatment in terms of the effective rate, WST, VFSS, IFRS, and IAP of dysphagia after stroke. Strict evaluation standards and high-quality RCT designs are necessary for further exploration.

## 1. Introduction

Dysphagia is a common disorder that occurs in approximately 34.7%–44% of stroke cases [1]. Poststroke dysphagia (PSD), characterized by swallowing difficulty in the oropharyngeal phase, can lead to many complications, such as dehydration, malnutrition, aspiration, and aspiration pneumonia [2]. It has been reported that patients with PSD have a 3-fold higher risk of aspiration pneumonia and a 5.4-fold higher mortality rate than patients without dysphagia [2]. For patients who experience PSD, the dysfunction can seriously affect their quality of life (QOL) by leading to social anxiety, withdrawal, and depression [3]. Thus, PSD creates a large financial burden on the families of stroke patients. Clinically, PSD treatment is mainly based on nondrug therapies, such as swallowing rehabilitation training (SRT), compensation therapy, physiotherapy, and alternative therapy [4]. However, most treatments only have a temporary and relatively limited effectiveness, and patients experiencing dysphagia after a stroke may seek other approaches.

Among the complementary and alternative therapies, electroacupuncture (EA) is an extension technique of acupuncture based on traditional acupuncture combined with modern electrotherapy, and it has been regarded as a promising method to treat PSD. A literature search yielded many published, clinical randomized controlled trials (RCTs) of EA for PSD. Evidence is the core of evidence-based medicine, and systematic reviews (SRs)/meta-analyses (MAs) based on RCTs are currently recognized as the highest level of evidence [5]. SRs/MAs are considered the gold standard for assessing the effects of health care interventions. The efficacy of acupuncture alone for PSD has been established using SRs/MAs [6]; however, the evidence of EA for PSD has not been assessed. Thus, we aimed to perform an SR/MA to investigate the efficacy and safety of EA for treating dysphagia in patients with stroke.

## 2. Materials and Methods

This SR/MA adheres to the guidelines for SRs/MAs according to the Cochrane Handbook [7] and the Preferred Reporting Items for Systematic Reviews and Meta-Analyses (PRISMA) guidelines [8]. The literature search, literature selection, data extraction, and quality evaluation were performed by both reviewers independently, and any inconsistencies were resolved through consensus or by consulting an experienced third reviewer.

### 2.1. Inclusion and Exclusion Criteria

The inclusion criteria were as follows: (a) study design: the trials had to be RCTs that aimed to compare combination therapy with swallowing training alone; (b) participants: participants had PSD diagnosed according to WHO criteria using appropriate radiological methods, not limited by gender, and age; (c) intervention: EA combined with SRT versus SRT alone; (d) outcomes: the primary outcome was an effective rate and swallowing function, as assessed by the water swallowing test (WST), video fluoroscopic swallowing study (VFSS), standardized swallowing assessment (SSA), and the Ichiro Fujishima rating scale (IFRS); the incidence of aspiration pneumonia (IAP) and adverse events were considered secondary outcomes. The exclusion criteria were as follows: (a) nonoriginal research articles; (b) studies in which the required data were unavailable.

### 2.2. Search Strategy

We searched the PubMed, Embase, Cochrane Library, Web of Science, the Chinese National Knowledge Infrastructure, Chinese Biomedical Database, and VIP databases from their inception to July 2020. The following keywords were used: dyssomnia, insomnia, auricular acupuncture, systematic review, and meta-analysis. The search strategy for the PubMed database is presented in Table 1, and it was be adjusted for each database.

### 2.3. Eligibility Assessment and Data Extraction

The titles and abstracts of all articles were screened first, and potentially eligible articles were retrieved for full-text reviews. The following data were collected from each RCT: first author; publication year; number of eligible cases; age; time after onset; intervention; and data related to effective rate, swallowing function assessment, adverse events, and QOL.

### 2.4. Quality Assessment

The methodological quality of the extracted trials was independently assessed by the two reviewers using the Cochrane risk of bias tool [6]. The methodological quality was assessed based on the following aspects: (1) random sequence generation; (2) allocation concealment; (3) blinding of participants and personnel; (4) blinding of outcome assessment; (5) incomplete outcome data; (6) selective reporting; and (7) other bias. Each domain includes one entry assigned a judgment of “Low risk,” “High risk,” or “Unclear risk” of bias.

### 2.5. Statistical Analyses

Review Manager Software 5.3.0 was used to perform MA of the included RCTs. Data were categorized as continuous and dichotomous variables. Continuous variables were analyzed using mean differences (MD) with 95% confidence intervals (CIs) or standardized mean differences (SMDs) if different measurement scales were used. Dichotomous variables were analyzed using odds ratios (ORs) with 95% CIs. Heterogeneity across studies was tested by the *I*^2^ statistic. The fixed effects model was used to analyze pooled data if heterogeneity was low (*I*^2^ < 50%); otherwise, the random effects model was used to analyze pooled data (*I*^2^ ≥ 50%). A funnel plot was used to examine the publication bias if 10 or more studies were pooled.

## 3. Results

### 3.1. Results on Literature Search and Selection

**Figure 1:**
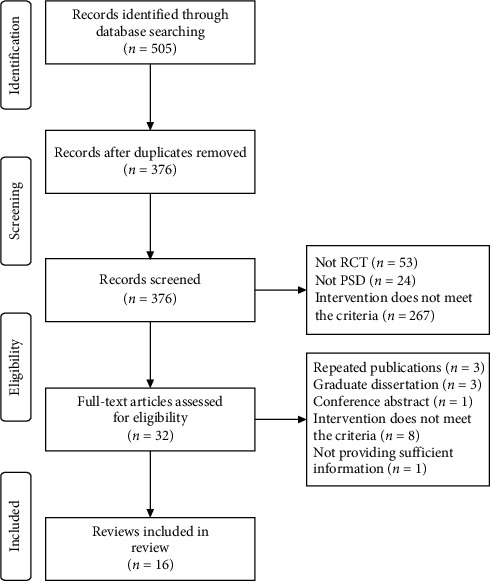
Flow chart of the literature selection process.

The search yielded 505 potential articles for review, 344 of which were excluded for reasons of irrelevance (Figure 1). Thirty-two clinical trials assessing the application of EA for PSD were retrieved for further assessment. Among these 32 trials, 16 were excluded because the intervention did not meet the inclusion criteria; they did not provide sufficient information; or they were duplicate publications, graduate dissertations, or conference abstracts. After excluding these 16 trials, the remaining 16 trials [9–24] were included in our review

### 3.2. Characteristic Summary of the Included Studies

In total, 16 RCTs involving 1,216 patients with PSD were included in this current review. The trials included were published between 2008 and 2019, the sample size ranged from 40 to 128, the ages of patients ranged from 36 to 79 years, and the duration of the treatment course ranged from 2 weeks to 1 month. All studies compared EA plus SRT with SRT alone. The outcome measures included both patient-centered outcomes and other routine test results. Two trials reported the incidence of aspiration pneumonia, and two trials reported adverse events. The detailed characteristics of the included studies are shown in Table 2.

### 3.3. Risk of Bias in Included Studies

**Figure 2:**
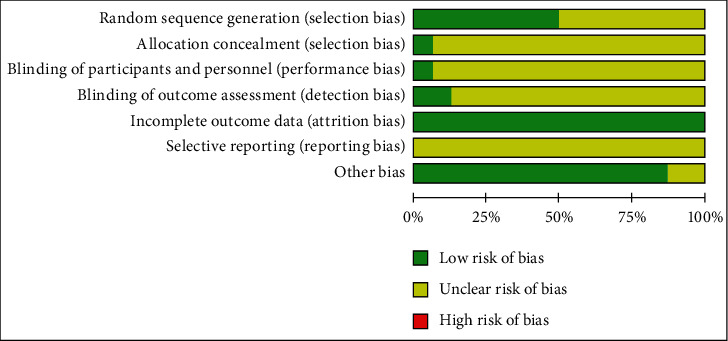
Risk of bias graph.

**Figure 3:**
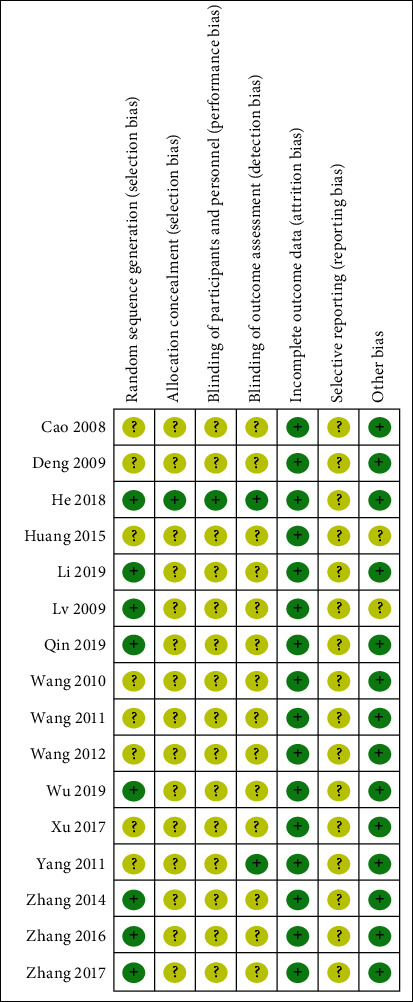
Risk of bias summary.

We assessed the risk of bias in all the included trials. Randomization was mentioned in all the trials, but only half of the studies described a specific method of randomization. Allocation concealment was inadequate in most trials (*n* = 15, 92.75%). Only one trial (*n* = 1, 6.25%) blinded their participants or personnel. Also, only one trial (*n* = 1, 6.25%) masked their outcome assessors to the treatment allocation, whereas a risk of selective reporting bias was not reported in all trials (*n* = 11, 100%). More details are shown in Figures 2 and 3.

### 3.4. Primary Outcome Measures

#### 3.4.1. Effective Rate

**Figure 4:**
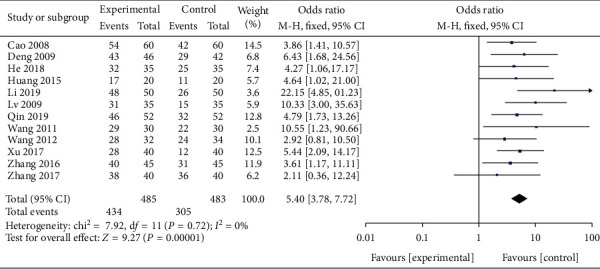
A forest plot for effective rate from 12 RCTs.

**Figure 5:**
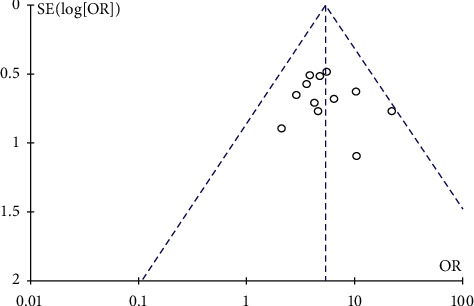
The funnel plot of the clinical efficacy rate.

The effective rate was reported by 12 trials, with 485 participants in the investigational group and 483 in the control group. All 12 RCTs were included in this meta-analysis to investigate the efficacy of EA for treating dysphagia in stroke patients. The meta-analysis was conducted using a random effects model. As shown in Figure 4, our meta-analysis revealed that patients who received EA combined with SRT for PSD had significantly greater benefits than SRT alone in terms of the effective rate (OR 5.40, 95% CI [3.78, 7.72], *P* < 0.00001, *I*^2^ = 0% (Figure 4). Furthermore, there was no publication bias in the funnel plot (Figure 5).

### 3.5. Second Outcome Measures

#### 3.5.1. Water Swallowing Test (WST)

**Figure 6:**
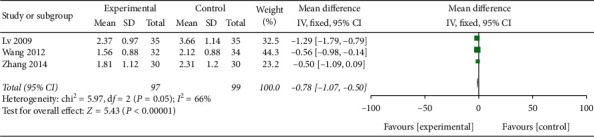
A forest plot for WST from 3 RCTs.

The WST was used in 6 trials; 3 of them used continuous data and were included in this meta-analysis. The meta-analysis was conducted using a random effect model, and the results showed that patients who received EA combined with SRT for PSD showed significantly greater benefits on the WST than those who received SRT alone (MD −0.78, 95% CI [−1.07, −0.50], *P* < 0.00001 (Figure 6)). There was substantial heterogeneity (*P*=0.05 (*I*^2^ = 66%)); after removing one trial [22], the heterogeneity decreased from 66% to 0%. The sensitivity analysis indicated that the source of the heterogeneity might be the post-onset time of this trial, which was longer than that of other trials.

#### 3.5.2. Video Fluoroscopic Swallowing Study (VFSS)

**Figure 7:**
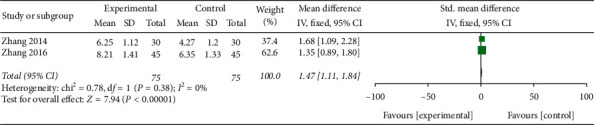
A forest plot for VFSS from 2 RCTs.

The VFSS was used in 3 trials; 2 of them were included in this meta-analysis. The meta-analysis was conducted using a random effects model, and the results showed that patients who received EA combined with SRT for PSD showed significantly greater benefits on the VFSS than those who received SRT alone (MD 1.47, 95% CI [1.11, 1.84], *P* < 0.00001, *I*^2^ = 0% (Figure 7)).

#### 3.5.3. Ichiro Fujishima Rating Scale (IFRS)

**Figure 8:**
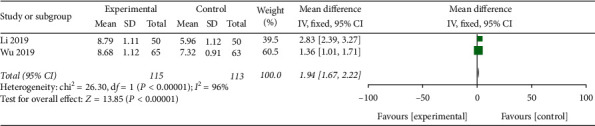
A forest plot for IFRS from 2 RCTs.

The IFRS was used in 2 trials, and both of them were included in this meta-analysis. The meta-analysis was conducted using a random effects model, and the results showed that patients who received EA combined with SRT for PSD showed significantly greater benefits on the IFRS than those who received SRT alone (MD 1.94, 95% CI [1.67, 2.22], *P* < 0.00001, *I*^2^ = 96% (Figure 8)).

#### 3.5.4. Incidence of Aspiration Pneumonia (IAP)

**Figure 9:**
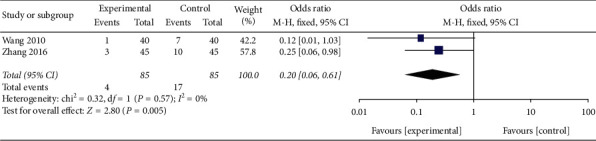
A forest plot for IAP from 2 RCTs.

The IAP was reported in 2 trials, and both of them were included in this meta-analysis. The meta-analysis was conducted using a random effect model, and the results showed that patients who received EA combined with SRT for PSD showed significantly greater benefits on the IAP than those who received SRT alone (OR 0.20, 95% CI [0.06, 0.61], *P*=0.005, *I*^2^ = 0% (Figure 9)).

#### 3.5.5. Adverse Events

Adverse events were mentioned in 2 trials [11, 12] and were reported by a total of 16 patients. Of these patients, 10 had adverse events related to acupuncture, such as pain and hematoma, but they were not severe. The remaining 6 patients developed irritating cough during eating, and they were all in the control group and received only SRT.

## 4. Discussion

EA and SRT are routine therapies based on traditional Chinese medicine or modern Western medicine and are commonly used to treat PSD. SRT or other therapies combined with EA have been used to treat PSD by physicians aiming to increase the therapeutic effectiveness and reduce the side effects. However, whether combination therapy has a more positive impact on PSD than conventional SRT alone is unclear. Therefore, the current MA was conducted to quantitatively evaluate the existing evidence on the efficacy and safety of the combination of EA and SRT for the treatment of PSD. A previous SR published in Chinese compared EA with acupuncture/SRT and EA plus SRT with SRT/medicine [25]. Our study differed in that we focused more specifically on EA in combination with SRT to explore the effect of this combination.

### 4.1. Summary of the Main Findings

First, this SR/MA of clinical RCTs published in the past 12 years was conducted to assess the benefits of EA among patients with PSD. Overall, our meta-analysis suggested that EA combined with SRT was significantly superior to SRT alone with regard to the effective rate, WST, VFSS, IFRS, and IAP. EA treatment was not associated with an increased risk of adverse events. However, the credibility of the results is limited due to the generally poor methodological quality of the included trials. Insufficient or inexact reports on allocation concealment, blinding of performance, and assessment were found in most of the included studies. In addition, the sample size of the included RCTs was generally small, and no trial used statistical methods to calculate the sample size before the trial; therefore, it is difficult for this MA to draw robust conclusions. Second, the assessment of the swallowing ability mainly included bedside and instrumental evaluation, and the VFSS is regarded as the “gold standard” for the diagnosis of dysphagia after stroke. However, other scales and questionnaires are more widely used in clinical practice to evaluate dysphagia after stroke due to the potential risks of aspiration, inconvenience of operation, and consideration of cost-effectiveness, while the diagnostic accuracy varies owing to the sensitivity and the specificity of the bedside assessment tools, and the subjective skills of the assessors. The scales of measurement that were used in the enrolled RCTs in our MA included the WST, IFRS, and VFSS, and all of them are subjective clinical assessment tools based on the observation of the assessors. Furthermore, the scale used to determine the clinical effective rate, which was classified as cured, markedly effective, effective, and ineffective, is not internationally recognized; hence, it may not be accurate for the assessment of the effect of treatment. The statistical analysis also demonstrated that the nonquantitative and symptom-descriptive measurements were the main sources of heterogeneity in this MA. This prevented us from drawing robust conclusions from the MA. Third, the long-term effects of this therapy could not be evaluated. The included studies only evaluated the use of EA for 2–4 weeks, and the outcomes were assessed before and immediately after treatment; thus, the long-term effects of EA on dysphagia after stroke could not be revealed. In addition, the protocols of EA are also diverse, including differences in acupoints, stimulation methods, needle retention time, and number of treatments, leading to sources of heterogeneity in this MA. Finally, there may be language bias, as all the included trials were conducted and published by Chinese investigators, which limits the general applicability of the study's conclusions outside of China.

### 4.2. Potential Mechanism of Action

PSD is a neuropathic swallowing disorder closely related to neuromuscular movement, and stroke leads to neuromuscular blockage involved in swallowing activities, which causes abnormalities in swallowing function. Our findings suggest that EA is beneficial to dysphagia after stroke. Modern studies have demonstrated the basic principle of EA in the treatment of PSD, and it is believed that the central nervous system has plasticity and reorganization ability, both structurally and functionally [2]. As an extension technique of acupuncture based on traditional acupuncture combined with modern electrotherapy, EA not only adheres to the theory of traditional Chinese medicine but also combines the physiotherapy mechanism of electrical stimulation. EA can directly act on the meridians and acupoints of the human body, promote the coordinated movement of multiple groups of muscle groups in the pharynx, and restore the muscle strength of damaged muscle groups [14]. Changes in EA current waveforms can present rhythmic contraction of muscles near the throat and prevent muscle waste atrophy after stroke [18]. Mechanical stimulation of EA at the lesion site can improve nerve excitability, reconstruct the damaged reflex arc, repair the injured nerve function, and then achieve a therapeutic effect [26]. Furthermore, EA increases blood perfusion in the medulla oblongata and some higher centers, which in turn improves pathological conditions, such as cerebral ischemia and hypoxia, and awakens inhibited nerve cells [12]. Thus, EA has been regarded as a promising method to treat PSD.

### 4.3. Strength and Limitations

To the best of our knowledge, this current study is the first SR/MA to explore the evidence of EA in combination with SRT for PSD. Based on the current results, it may have a certain reference value for the clinical practice and research of EA in the treatment of PSD. Of the 16 included trials, 75% used the effective rate to assess the effectiveness of EA for PSD, and the definition of the effective rate was as follows: effective rate = (“total number of patients” − “number of patients with no response”)/total number of patients, and “no response” meant no significant change in any aspect of the swallowing function or regression of one aspect of the swallowing function after treatment. However, most of these trials did not use consistent measurement tools to assess the changes in the swallowing function before and after the treatment to reflect the effective rate; therefore, the credibility of the effective rate is limited. Hence, the swallowing function assessed by the gold standard of VFSS should be considered as the primary outcome for future research.

## 5. Conclusion

The findings of this quantitative MA showed that EA combined with SRT treatment for patients with PSD resulted in an additional benefit on the effective rate. Given the low quality of the included trials, these results are not conclusive, and further high-quality and large-scale RCTs are required to verify the therapeutic effects of EA on PSD.

## Figures and Tables

**Table 1:** Search strategy for the PubMed database.

Query	Search term
#1	Cerebrovascular disorders [Mesh] OR stroke [Mesh] OR brain infarction [Mesh] OR cerebral hemorrhage [Mesh]
#2	Cerebrovascular disorder*∗* [Title/Abstract] OR stroke*∗* [Title/Abstract] OR brain infarction*∗* [Title/Abstract] OR cerebral hemorrhage*∗* [Title/Abstract] OR intracranial vascular disease*∗* [Title/Abstract] OR cerebrovascular disease*∗* [Title/Abstract] OR brain vascular disorder*∗* [Title/Abstract] OR cerebrovascular occlusion*∗* [Title/Abstract] OR cerebrovascular insufficiency*∗* [Title/Abstract] OR cerebrovascular accident*∗* [Title/Abstract] OR cerebrovascular apoplexy [Title/Abstract] OR brain vascular accident*∗* [Title/Abstract] OR apoplexy [Title/Abstract] OR anterior cerebral circulation infarction [Title/Abstract] OR cerebrum hemorrhage*∗* [Title/Abstract] OR intracerebral hemorrhage*∗* [Title/Abstract] OR brain hemorrhage [Title/Abstract]
#3	#1 OR #2
#4	Deglutition disorders [Mesh]
#5	Deglutition disorder*∗* [Title/Abstract] OR swallowing disorder*∗* [Title/Abstract] OR dysphagia [Title/Abstract] OR oropharyngeal dysphagia [Title/Abstract] OR esophageal dysphagia [Title/Abstract]
#6	#4 OR #5
#7	Acupuncture [Mesh]
#8	Acupuncture [Title/Abstract] OR acupuncture therapy [Title/Abstract] OR electroacupuncture [Title/Abstract] OR electro acupuncture [Title/Abstract] OR electric acupuncture [Title/Abstract] OR electrical acupuncture [Title/Abstract] OR electrical stimulation therapy [Title/Abstract]
#9	#7 OR #8
#10	Randomized controlled trials as topic [Mesh]
#11	Randomized controlled trials [Title/Abstract] OR random*∗* [Title/Abstract] OR controlled clinical trial [Title/Abstract] OR rct [Title/Abstract]
#12	#10 OR #11
#13	#3 AND #6 AND #9 AND #12

**Table 2:** Characteristics of the included studies.

First author; year	No. of patients		Age		Time after onset		Therapy duration	Outcomes	Intervention	
	*I*	*C*	*I*	*C*	*I*	*C*			*I*	*C*
Wu et al. [9] 2019	63	65	44.0 ± 2.9	44.3 ± 2.6	3.9 ± 0.1 w	3.7 ± 0.4 w	3 w	IFRS	EA + SRT	SRT
Li et al. [10] 2019	50	50	42.5 ± 2.3	42.5 ± 2.2	26.9 ± 1.6 d	25.7 ± 1.5 d	20 d	IFRS, WST, ER	EA + SRT	SRT
Qin et al. [11] 2019	52	52	53.4 ± 10.7	53.8 ± 11.4	6.2 ± 1.5 m	6.4 ± 1.4 m	4 w	SSA, WST, ER, AE	EA + SRT	SRT
He et al. [12] 2018	35	35	64 ± 6	69 ± 7	32 ± 15 d	27 ± 15 d	4 w	ER, AE	EA + SRT	SRT
Xu et al. [13] 2017	40	40	53.98 ± 5.44	55.43 ± 5.67	9.96 ± 1.47 d	10.34 ± 1.54 d	1 m	ER	EA + SRT	SRT
Zhang et al. [14] 2017	40	40	51∼75	53∼76	6∼28 d	7∼27 d	4 w	VFSS,WST, ER	EA + SRT	SRT
Zhang et al. [15] 2016	45	45	62.4 ± 9.6	61.2 ± 10.1	12.8 ± 4.6 d	11.6 ± 4.4 d	4 w	VFSS, ER, IAP	EA + SRT	SRT
Huang and Yang [16] 2015	20	20	50∼70	50∼70	<72 h	<72 h	2 w	ER	EA + SRT	SRT
Zhang [17] 2014	30	30	54.2 ± 4.3	53.7 ± 2.9	3.9 ± 0.5 m	3.5 ± 0.9 m	2 w	WST, VFSS	EA + SRT	SRT
Wang et al. [18] 2012	32	34	63.8 ± 9.3	68.1 ± 10.3	4d∼7 m	3d∼6 m	30 d	WST, ER	EA + SRT	SRT
Wang et al. [19] 2011	30	30	36∼79	39∼73	14∼78 d	14∼78 d	4 w	ER	EA + SRT	SRT
Yang et al. [20] 2011	35	35	67.9 ± 10.6	67.4 ± 9.8	8.3 ± 11.3 d	9.6 ± 15 d	3 w	SSA	EA + SRT	SRT
Wang and Cheng [21] 2010	40	40	67.4 ± 7.8	68.3 ± 9.3	Unclear	Unclear	30 d	IAP	EA + SRT	SRT
Lv et al. [22] 2009	35	35	52∼75	52∼75	0.2∼10 m	0.2∼10 m	2 w	ER, WST	EA + SRT	SRT
Deng and Wang [23] 2009	46	42	42∼79	45∼76	<10 d	<10 d	30 d	ER	EA + SRT	SRT
Cao [24] 2008	60	60	47∼70	51∼68	Unclear	Unclear	4 w	ER	EA + SRT	SRT

C: control group; I: intervention group; EA: electroacupuncture; SRT: swallowing rehabilitation training; WST: water swallow test; ER: effective rate; SSA: standardized swallowing assessment; AE: adverse events; VFSS: video fluoroscopic swallowing study; IAP: incidence of aspiration pneumonia; IFRS: Ichiro Fujishima Rating Scale.

## Data Availability

The data used to support the findings of this study are available from the corresponding author upon request.

## Abbreviations

EA: Electroacupuncture
PSD: Poststroke dysphagia
RCT: Randomized controlled trial
MA: Meta-analysis
SRT: Swallowing rehabilitation training
WST: Water swallowing test
ER: Effective rate
SSA: Standardized swallowing assessment
AE: Adverse events
VFSS: Video fluoroscopic swallowing study
IAP: Incidence of aspiration pneumonia
IFRS: Ichiro Fujishima Rating Scale
QOL: Quality of life
SR: Systematic review
PRISMA: Preferred Reporting Items for Systematic Reviews and Meta-Analyses
MD: Mean differences
CI: Confidence intervals
SMD: Standardized mean difference
OR: Odds ratio.
